# Antipsychotic drug-induced neutropenia: results from the AMSP drug surveillance program between 1993 and 2016

**DOI:** 10.1007/s00702-023-02589-7

**Published:** 2023-01-18

**Authors:** Catherine Glocker, R. Grohmann, G. Burkhardt, J. Seifert, S. Bleich, T. Held, S. Toto, S. Stübner, C. Schüle

**Affiliations:** 1 Department of Psychiatry und Psychotherapy, LMU Klinikum, Nußbaumstraße 7, 80336 Munich, Germany; 2 Department of Psychiatry, Social Psychiatry and Psychotherapy, Hannover Medical School, Carl-Neuberg-Str. 1, 30625 Hannover, Germany; 3 Department of Hematology and Cell Therapy, Helios Klinikum Berlin-Buch, Schwanebecker Chaussee 50, 13125 Berlin, Germany; 4 Department of Forensic Psychiatry, Bezirksklinikum Ansbach, Feuchtwanger Str. 38, 91522 Ansbach, Germany

**Keywords:** Adverse drug reaction, Antipsychotic drug-induced neutropenia, Agranulocytosis, AMSP program, Psychiatric inpatients

## Abstract

Neutropenia and agranulocytosis (N&A) are relatively rare, but potentially fatal adverse drug reactions (ADR). This study presents cases of N&A related to one or more antipsychotic drugs (APDs) in psychiatric inpatients. Data on APD utilization and reports of N&A caused by APDs were analyzed by using data from an observational pharmacovigilance program in German-speaking countries—Arzneimittelsicherheit in der Psychiatrie (AMSP)—from 1993 to 2016. 333,175 psychiatric inpatients were treated with APDs for schizophrenia and other indications during the observation period. A total of 124 cases of APD-induced N&A were documented, 48 of which fulfilled the criteria for agranulocytosis, corresponding to a rate of 0.37, respectively, 0.14 in 1000 inpatients treated with APDs. Neutropenia was more often detected in women, whereas there was no difference regarding sex in cases of agranulocytosis. Clozapine had the highest relative risk for inducing N&A and was imputed alone as a probable cause of N&A in 60 cases (1.57‰ of all patients exposed). Perazine showed the second highest relative risk with 8 cases and an incidence 0.52‰, followed by quetiapine (15 cases resp. 0.23‰ of all patients exposed) and olanzapine (7 cases; 0.13‰ of all patients exposed). N&A most often occurred during the first 3 months of treatment. Overall N&A are severe and potentially fatal complications that can occur during treatment with APDs. The results from this study largely agree with the currently available literature, highlighting the positive effects of alertness and established appropriate monitoring.

## Introduction

A healthy human adult has 4.5–10 billion white blood cells/l, of which neutrophils account for about 60% (Curtis 2017). Neutrophils are critical in defense against infectious pathogens such as bacteria and fungi (Curtis 2017). A reduction of neutrophils in the blood to an absolute neutrophil count (ANC) of < 1.5 × 10^9^ cells/l is defined as neutropenia. Neutropenia is considered to be severe when the ANC falls below 0.5 × 10^9^ cells/l, a condition often called agranulocytosis*.* The majority of patients with agranulocytosis have an ANC < 0.1 × 10^9^/l. Patients initially presenting with an ANC < 0.1 × 10^9^/l have been shown to have a greater risk of more severe complications including sepsis and death compared to those with a higher ANC (Andersohn et al. 2007; Curtis 2017)*.* Drug-induced neutropenia and agranulocytosis (N&A)—not associated with chemotherapy—are relatively rare but potentially fatal adverse drug reactions (ADR) that occur in susceptible individuals with an incidence of approximately 1.6–15.4 cases per million population per year (Curtis 2014, 2017). Although the pathogenesis is not fully elucidated, two mechanisms appear to primarily contribute to the occurrence of this ADR: (1) direct toxicity to the myeloid cell line and (2) immune-mediated destruction (Pick and Nystrom 2014). Drugs most often associated with neutropenia or agranulocytosis include spironolactone, carbamazepine, antibiotics (beta-lactam and cotrimoxazole), antiplatelet agents (ticlopidine), antithyroid drugs, sulfasalazine, nonsteroidal anti-inflammatory agents and clozapine (Andrès and Maloisel 2008; Curtis 2014). Patients suffering from drug-induced neutropenia typically experience severe neutropenia within several weeks to several months after initial exposure to the drug (Curtis 2017). A systematic review by Andersohn et al. (2007) found that the median duration of drug exposure before the onset of neutropenia ranges from 2 to 60 days. In our previous study “Blood dyscrasias induced by psychotropic drugs” (Stübner et al. 2004) we found a clear peak of severe neutropenias between the 20th and 30th day after onset of treatment. The time course of the clozapine-induced changes, however, showed another pattern: the main peak appeared later, i.e., between the 40th and 50th day of treatment.

Patients with acute, severe neutropenia or agranulocytosis may experience symptoms such as fever, sore throat, acute tonsillitis, muscle and joint pain, septicemia and pneumonia (Andrès et al. 2017; Curtis 2017). However, patients affected with non-chemotherapy drug-induced N&A are often asymptomatic, making this ADR difficult to detect. The early detection and timely correction of neutrophils to normal levels are critical in preventing severe infections and death (Curtis 2017; Pick and Nystrom 2014)*.* Therefore, differential blood counts should be taken regularly as this is crucial especially when patients are prescribed drugs with a high risk of inducing N&A.

Mortality of drug-induced N&A is currently estimated at 5% (Pick and Nystrom 2014), which is significantly lower than assessments 20 years ago, which were as high as 20% (Rao 2014)*.* This finding is probably a result of improved education of both patients and physicians regarding the risk of drug-induced N&A, especially for commonly implicated drugs (e.g., clozapine). Regular monitoring of the blood count allows for early recognition and timely initiation of optimum therapy (Curtis 2017).

Psychotropic drugs have been associated with blood dyscrasias including N&A since the early days of their use (Hiob and Hippius 1955), and since the introduction of clozapine, this ADR has received more attention (Duggal and Singh 2005). Almost all the major classes of psychotropic drugs have been associated with neutropenia. Clozapine is the drug that is most often associated with non-chemotherapy drug-induced N&A. In 1974, eight fatal cases of agranulocytosis in Finland led to the suspension of clozapine and demonstrated the need for a mandatory hematologic monitoring program (Idänpään-Heikkilä et al. 1977; Nielsen et al. 2013). The risk of clozapine-induced agranulocytosis is 0.7%, while the risk of neutropenia is significantly higher (approximately 3%) (Nielsen et al. 2013). The current mandatory monitoring regimen has made fatal agranulocytosis extremely rare with incidences as low as 0–0.03% (Munro et al. 1999; Nielsen et al. 2013). The risk of agranulocytosis appears to be highest during the first 3–6 months of treatment, but neutropenia may occur at any time (Pick and Nystrom 2014). Occurrence of neutropenia typically provokes concern that the absolute neutrophil count will continue to drop and reach the agranulocytosis level, a development that is unpredictable (Nielsen et al. 2013)*.* Phenothiazines (e.g., chlorpromazine) have been shown to cause benign leukopenia in up to 10% of patients, whereas the occurrence of agranulocytosis is rare (Duggal and Singh 2005)*.* With increased awareness of drug-induced N&A among physicians, monitoring protocols facilitating early detection and modern treatment methods of this ADR have attracted attention within recent literature (Duggal and Singh 2005).

Management of drug-induced agranulocytosis includes the immediate discontinuation of the offending drug(s) and initiation of broad-spectrum antibiotics. In patients concomitantly treated with multiple drugs, it can be difficult to determine the offending drug, but primary consideration should be given to those medications frequently associated with drug-induced neutropenia (Curtis 2017). After drug discontinuation, most cases of N&A resolve within 9 days (range 2–24 days) (Andrès et al. 2010) and require only symptomatic therapy such as antibiotics for treatment and prophylaxis of infections and good hygiene practices (Pick and Nystrom 2014). The use of granulocyte colony-stimulating factors (G-CSF) in high-risk patients may also be considered (Pick and Nystrom 2014), although this treatment is controversial (Curtis 2017). Some reports show that the use of G-CSF is associated with a shorter duration of N&A (Andrès et al. 2017), antibiotic therapy and length of hospitalization (Pick and Nystrom 2014). It has also been reported that patients treated with hematopoietic growth factors, when asymptomatic at diagnosis, had a lower incidence of infectious or fatal complications than patients not receiving this treatment (Andersohn et al. 2007).

This study presents cases of N&A related to one or more antipsychotic drugs (APDs) in psychiatric inpatients. The relative frequencies of the examined ADRs were estimated for drugs most commonly used. Drawing on the large dataset of the European drug surveillance program “Drug Safety in Psychiatry” (German: „Arzneimittelsicherheit in der Psychiatrie“; AMSP), the present naturalistic study updates previous contributions [“Agranulocytosis and significant leucopenia with neuroleptic drugs” (Grohmann et al. 1989) and “Blood dyscrasias induced by psychotropic drugs” (Stübner et al. 2004)].

## Methods

Data on severe ADRs and psychotropic drug utilization have been collected by the AMSP program since 1993. AMSP is a continuous drug surveillance program that permits pharmacovigilance in a naturalistic setting. It is especially designed to evaluate severe ADRs to psychotropic drugs in psychiatric inpatients. AMSP generates an ongoing database of severe ADRs occurring in inpatients within psychiatric hospitals in Germany, Austria and Switzerland.

This study includes data from 1993 to 2016, presenting a sample twice as large as in our previous publication on this ADR (Stübner et al. 2004). AMSP assesses severe ADRs (Grohmann et al. 2004) that occur during routine clinical treatment. Trained psychiatrists act as drug monitors that collect data on ADRs and document these cases using a standardized questionnaire. After review by a senior member of AMSP, the cases are discussed at central case conferences in which drug monitors from participating hospitals gather together with representatives of the Federal Health Agency (BfArM) and the Drug Commission of the German Medical Association (AkdÄ), as well as drug safety experts from the pharmaceutical industry. Here, the final judgment on the imputation of one or more drugs concerning the observed ADR is made including a probability rating of each drug assumed to be involved in the ADR (Grohmann et al. 2004).

- *Grade 1* possible (ADR unknown or alternative explanation more likely).
- *Grade 2* probable (ADR known for drug in question and time course and dosage in accordance with previous experience; alternative explanation less probable).
- *Grade 3* definite (the same as G*rade 2* with re-occurrence of the ADR after re-exposure to the drug in question).
- *Grade 4* questionable or not sufficiently documented.

When an agreement has been reached and probability ratings have been given to the imputed drugs, the case questionnaires are sent to the relevant authorities and pharmaceutical companies and saved in a fully anonymized manner at the central database of the AMSP for future analysis.

In case of polypharmacy, multiple drugs are often imputed. When a pharmacodynamic interaction is held responsible for an ADR, each of the imputed drugs is given a rating of “possible”, “probable” or “definite” according to the given facts. In this report we only refer to ADRs in which involvement of the drug(s) in question has been rated as “probable” or “definite” (grade 2 and 3).

The AMSP database evaluates cases of ADRs from two different perspectives. The first perspective considers all ADRs in which one or more drugs were causally involved, therefore also including ADRs associated with polypharmacy (referred to as “all cases”). The second perspective only focuses on ADRs, in which a single drug/drug class was causally involved (referred to as “imputed alone”).

The definition of a clinically severe ADR is given in a detailed study protocol (Grohmann et al. 2004). Neutropenia is defined as < 1.5/nl and agranulocytosis as < 0.5/nl. AMSP includes neutropenia as well as agranulocytosis as a severe ADR.

Data on drug use at the participating hospitals are assessed on two reference days per year on which all administered drugs and their doses are documented for all psychiatric inpatients along with basic demographic and diagnostic data. Moreover, the contributing hospitals provide the number of inpatients and the mean duration of inpatient care for all patients under surveillance per year so that relative frequencies can be calculated.

### Ethics review

Evaluations using the AMSP database have been approved by the Ethics Committee of the University of Munich and the Ethics Committee of the Hannover Medical School (Nr. 8100_BO_S_2018). This study adheres to the Declaration of Helsinki and its later amendments. The AMSP program is a continuous observational post-marketing drug surveillance program and does not interfere with the ongoing clinical treatment of the patients under surveillance. Furthermore, evaluation data were obtained from the anonymized data bank and individual patients cannot be traced.

### Statistical analysis

Statistical analyses were conducted using the program “R” (Dessau and Pipper 2008). The incidence of APD-induced N&A was calculated in relation to the number of patients exposed to a given drug, drug group or subgroup and is reported along with its 95% confidence interval (CI). Due to the low rate of N&A, only drugs used in 5000 or more patients were included in statistical analyses. With regard to the very low rate of serious ADRs and the high number of individuals exposed, confidence intervals were calculated using the exact method (Vollset 1993), avoiding the bias of commonly used approximate methods (Agresti and Coull 1998). Statistical comparisons of the incidence of N&A related to diagnoses, sex and age were performed by means of Chi-square tests. Due to low rates of non-schizophrenia diagnoses among ADR cases, these were grouped together (schizophrenia vs others) for further analysis. For the evaluation according to age, the following intervals were chosen: 18–64 years, ≥ 65 years.

## Results

### Sociodemographic and illness-related data

A total of 495,615 psychiatric inpatients were monitored within the AMSP program between 1993 and 2016. During this time frame, 333,175 patients were treated with APDs. Patients treated with APDs most often suffered from a primary diagnosis of schizophrenia, schizotypal and delusional disorders, followed by mood and organic mental disorders. A total of 124 cases of APD-induced N&A were documented, 48 of which fulfilled the criteria for agranulocytosis. This corresponds to a rate of 0.37 and 0.14 cases, respectively, in 1000 inpatients treated with APDs. The risk of neutropenia appeared to differ according to sex. While 41 cases of N&A occurred in men (33% of all cases, 0.27 per 1000 inpatients), about 2/3 of the cases (*n* = 83) affected women (67% of all cases; 0.46 per 1000 inpatients). We did not detect a sex-related difference in the incidence of agranulocytosis. Agranulocytosis was observed in male patients in 20 cases (0.13 per 1000 inpatients) and 28 cases in women, (0.15 per 1000 inpatients). The incidence in the different age groups (18–64 and ≥ 65 years) was identical for both N&A (0.37 per 1000 inpatients) and agranulocytosis only (0.14 per 1000 inpatients). Cases of N&A occurred significantly more often (*p* < 0.01) in patients treated with APDs diagnosed with schizophrenia, schizotypal and delusional disorders (0.61 resp. 0.26 per 1000) and mania (0.54 resp. 0.18 per 1000). Patients treated with APDs suffering from neurosis and personality disorders (0.22 per 1000, no cases of agranulocytosis), organic mental disorders (0.19 resp. 0.10 per 1000) or major depression (0.12 resp. 0.02 per 1000) were less commonly affected (Table 1).

**Table 1:** ICD-10 diagnosis, age, sex and monitored inpatients exposed to antipsychotic drugs (APD) (*N* = 333.175) compared to cases of neutropenia/agranulocytosis (N&A) (*N* = 124) and cases of agranulocytosis (*N* = 48)

	Monitored inpatients with APD, *n* (% of 333,175)	Cases with N&A, *n* (% of 124)	Incidence in‰ inpatient admissions	*p*
Diagnosis (ICD-10)				< 0.01
Schizophrenia (F2-)	153,850 (46.18)	93 (75.00)	0.61	
Mania (F30/F31.-)	11,149 (3.35)	6 (4.84)	0.54	
Organic mental disorders (F0-)	41,583 (12.48)	8 (6.45)	0.19	
Neurosis/personality disorders (F4-/F6-)	27,049 (8.12)	6 (4.84)	0.22	
Others (F1-/F5-/F7-)	13,771 (4.13)	1 (0.81)	0.07	
Age				0.9696
18–64	262,850 (78.89)	98 (79.03)	0.37	
≥ 65	70,325 (21.11)	26 (20.97)	0.37	
Sex				0.01
Male	150,591 (45.20)	41 (33.06)	0.27	
Female	182,584 (54.80)	83 (66.94)	0.46	

	Monitored inpatients with APD, *n* (% of 333,175)	Cases with agranulocytosis, *n* (% of 48)	Incidence in‰ inpatient admissions	
Diagnosis (ICD-10)				< 0.01
Schizophrenia (F2-)	153,850 (46.18)	40 (83.33)	0.26	
Depression (F31/F32/F33.-)	85,773 (25.74)	2 (4.17)	0.02	
Mania (F30/F31.-)	11,149 (3.35)	2 (4.17)	0.18	
Organic mental disorders (F0-)	41,583 (12.48)	4 (8.33)	0.10	
Age				0.72
18–64	262,850 (78.89)	38 (79.17)	0.14	
≥ 65	70,325 (21.11)	10 (20.83)	0.14	
Sex				0.96
Male	150,591 (45.20)	20 (41.67)	0.13	
Female	182,584 (54.80)	28 (58.33)	0.15	

### Antipsychotic drugs associated with neutropenia

14 different APDs were attributed to the total of 124 cases of N&A. In 101 cases (81.5% of all neutropenia cases) a single APD was held responsible for the ADR as the only probable cause. Only 22 of these cases (17.7%) occurred during monotherapy (14 cases under treatment with clozapine, 3 cases under perazine, 2 cases under quetiapine and 1 case each under treatment with haloperidol, olanzapine and aripiprazole). In 23 cases (18.6%), combinations of several drugs were imputed as equal probable contributors to N&A. In 45 cases (36.3%), other drugs (between one and three additional drugs per case) were imputed additionally as possible contributors to N&A. Most cases imputing more than one drug were due to the combination of two APDs. Non-antipsychotic drugs were imputed as probable cause for N&A in combination therapy in six cases: we found two cases in combination therapy with carbamazepine, two cases with antibiotics (one case with metronidazole/ceftriaxone, one case with only ceftriaxone), one case with the antidiabetic drug gliclazide and one case with the cytostatic drug cyclophosphamide. Sometimes additional drugs, like NSAR or antibiotics, were imputed as possible contributors (rating as grade 1) to the ADR.

**Fig. 1:**
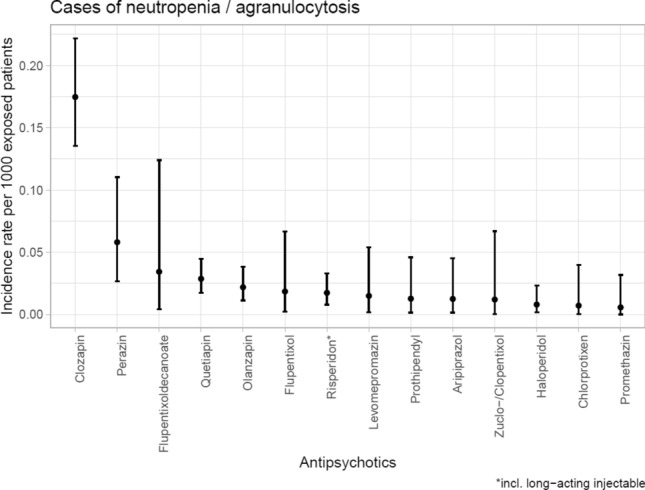
Incidence (95% confidence intervals) of neutropenia/agranulocytosis with imputed antipsychotic drugs

**Fig. 2:**
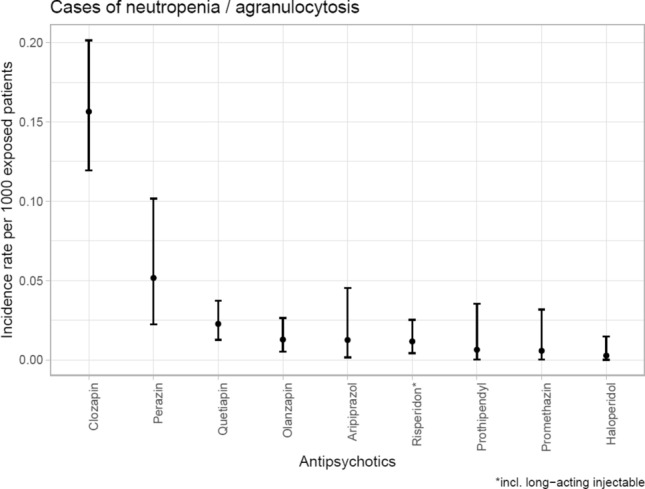
Incidence (95% confidence intervals) of neutropenia/agranulocytosis with imputed antipsychotic drugs (only imputed alone)

Table 2 and Figs. 1 and 2 show the incidence of N&A during treatment with different APDs. Most cases of N&A occurred during treatment with clozapine. Clozapine was the APD with the highest relative risk for inducing N&A and was considered causal in 67 cases (1.75‰ of 38,349 patients exposed). Clozapine was imputed alone in 60 cases (1.57‰ of all patients exposed). Perazine showed the second highest relative risk with nine cases and an incidence of 0.58‰ (eight cases and 0.52‰ when imputed alone) of 15,495 patients exposed. Quetiapine was imputed alone as probable cause in 15 cases (0.23‰ of 66,209 patients exposed), followed by olanzapine (7 cases; 0.13‰ of 54,822 patients exposed). Among subgroups of APDs, we found most cases of N&A to occur under treatment with second-generation APDs, which were consequently imputed alone in 91 cases or 0.40‰ of 226,161 patients exposed. Among first-generation APDs, most cases were associated with the use of phenothiazines (imputed alone in ten cases or 0.14‰ of 69,793 patients exposed).

**Table 2:** Incidence of neutropenia/agranulocytosis between 1993 and 2016 during antipsychotic drug (APD) use in relation to all patients exposed (*n* > 5000)

Imputed drug	Patients monitored, *n*	Number of cases, *n* (all cases)	Number of cases, *n* (imputed alone)	Incidence in‰ patients exposed (all cases)	Incidence in‰ patients exposed (imputed alone)
All APD	333,175	124	101	0.37	0.30
First-generation APD	172.134	21	11	0.12	0.06
High potency APD	92,980	16	10	0.17	0.11
Low potency APD	102,468	6	2	0.06	0.02
Butyrophenones	85,264	3	1	0.04	0.01
Haloperidol (incl. long-acting injectables)	40,511	3	1	0.07	0.03
Thioxanthenes	38,961	4	1	0.10	0.03
Flupentixol (incl. long-acting injectables)	14,979	4	0	0.27	0
Chlorprothixene	14,017	1	0	0.07	0
Zuclopentixol	8320	1	0	0.12	0
Phenothiazines	69,793	15	10	0.22	0.14
Prothipendyl	15,742	2	1	0.13	0.06
Perazine	15,495	9	8	0.58	0.52
Levomepromazine	13,374	2	0	0.15	0
Promethazine	17,546	1	1	0.06	0.06
Second-generation APD	226,161	108	91	0.48	0.40
Clozapine	38,349	67	60	1.75	1.57
Olanzapine	54,822	12	7	0.22	0.13
Quetiapine	66,209	19	15	0.29	0.23
Aripiprazole	15,988	2	2	0.13	0.13
Risperidone (incl. long-acting injectables)	53,948	9	6	0.17	0.11

Drugs used in less than 5000 patients were not included

Most of the 48 cases of agranulocytosis also occurred under treatment with clozapine, which accounted for 41 cases (85.4% of all cases of agranulocytosis) and was imputed alone in 35 cases (72.9% of all cases of agranulocytosis). Perazine was imputed alone as probable cause in two cases, prothipendyl, quetiapine and aripiprazole were each imputed alone in one case of agranulocytosis, whereas olanzapine (two cases) and levomepromazine (one case) were imputed in combination with other drugs.

Table 3 shows the median time to onset of APD-induced N&A after initiation of treatment with the imputed APDs. The median duration until the onset of APD-induced N&A was 29 days, while APD-induced agranulocytosis was detected after a median duration of 49 days. The earliest occurrence of neutropenia was seen under treatment with quetiapine after 2 days. The majority of cases of quetiapine-induced N&A were detected during the first 2 weeks of treatment and therefore earlier than neutropenia associated with any other APD. The latest case of APD-induced neutropenia was reported after almost 20 years of treatment with clozapine. APD-induced neutropenia was detected earlier than APD-induced agranulocytosis for all APDs with the exception of clozapine.

**Table 3:** Median duration between start of the imputed drugs and beginning of neutropenia/agranulocytosis; number of N&A/agranulocytosis cases after time intervals

Imputed drug	Median duration in days	Cases after < 2 weeks	Cases after 2–4 weeks	Cases after 4–8 weeks	Cases after 8–12 weeks	Cases after 12–18 weeks	Cases after 18 weeks–1 year	Cases after > 1 year
All drugs	29/49							
Aripiprazole	32/22		1/1	1/0				
Chlorprotixene	27		1/0					
Clozapine^a^	73/54	2/1	8/4	21/16	14/10	5/3	8/3	8/4
Flupentixol (incl. long-acting injectables)	19		2/0					
Haloperidol (incl. long-acting injectables)	15	1/0	2/0					
Levomepromazine	23/29		1/0	1/1				
Olanzapine^a^	16/35	4/0	2/0	3/2			1/0	1/0
Perazine^a^	30/26	2/0	3/2	1/0		1/0		
Promethazine	22		1/0					
Prothipendyl	51/80			1/0	1/1			
Quetiapine^a^	16/1341	8/0	1/0	4/0			2/0	2/1
Risperidone^a^ (incl. long-acting injectables)	21	3/0	3/0	1/0				1/0

^a^Lower case numbers due to missing data regarding the onset of ADRs

The median dosage of clozapine did not differ between patients who developed neutropenia or agranulocytosis and was also the same for all patients exposed (300 mg daily dosage each). The ADR also occurred at lower dosages of clozapine (50 and 75 mg per day in two cases each, 100 mg per day in three cases). The median dosage of perazine in cases of N&A was higher than the dosage of all patients exposed to the drug (350 resp. 400 mg vs. 150 mg/day). The same trend was observed in patients treated with quetiapine (250 mg resp. 800 mg in all neutropenia resp. only agranulocytosis cases vs. 200 mg/day in all patients exposed) and risperidone (4 vs. 3 mg/day). The median daily dosages in the cases of APD-induced N&A were lower than among all patients exposed to several first-generation APDs (i.e., haloperidol; see Table 4). Unfortunately, data on serum drug concentrations and starting dosage are lacking.

**Table 4:** Median daily dosages in monitored patients and all neutropenia/agranulocytosis (N&A) resp. agranulocytosis cases under treatment with imputed antipsychotic drugs (3 or more cases)

Antipsychotic drug	Number of all N&A cases	Number of agranulocytosis cases	Median dosage (mg/day), all patients exposed	Median dosage (mg/day) all N&A cases (Min./Max.)	Median dosage (mg/day) agranulocytosis cases (Min./Max.)
Clozapine	67	41	300	300 (50/800)	300 (50/800)
Haloperidol	3		6	1.5 (1.5/8)	
Olanzapine	12	2	15	15 (2.5/20)	
Perazine	9	2	150	350 (75/600)	
Quetiapine	19	1	200	250 (50/1350)	
Risperidone (incl. long-acting injectables)	8		3	4 (1.5/12)	

### Course of the ADR, risk factors and countermeasures

In 102 of the 124 cases (82.3%), the ADR fully subsided. In 16 cases (12.9%), N&A was in the process of subsiding, while it remained unchanged in 1 case by the end of observation. The further course of the ADR is unknown in two cases. A total of three patients died as a result of APD-induced agranulocytosis (2.4%). In most cases (i.e., 92 cases, 74.2%) no risk factors were documented. In 17 cases (13.7%), a general sensitivity to ADRs was found. Pre-existing damage of the hematopoietic system was documented in a total of seven cases (four cases with pre-existing decreased leukocytes, two cases of hematologic disease and one case of toxic damage to the hematopoietic system caused by alcohol). The imputed APD was discontinued in 121 cases (97.6%). In 30 cases (24.2%), patients were transferred to another department or hospital for specialized care. To treat the ADR (agranulocytosis in all of these cases) or its complications, drugs such as G-CSF and antibiotics were used in 28 cases (22.6%) and other countermeasures such as isolation were applied in 17 cases (13.7%).

### Symptomatology in agranulocytosis cases

In 20 of the 48 agranulocytosis cases (41.7%), the ANC was < 0.1 × 10^9^ cells/L and in 28 cases the ANC was between 0.10 and 0.49 × 10^9^ cells/L. Only 19 (39.6%) of the patients with APD-induced agranulocytosis presented with clinical symptoms such as fever, herpes, swelling of the lymph nodes, sore throat, angina tonsillaris, deterioration of the general state of health, pneumonia, sepsis, nausea, vomiting, diarrhea, somnolence, abscesses, cystitis, urticaria, skin eruptions, mild symptoms of a common cold and aphthae. All three fatal cases were of females over 65 years of age. Two fatal cases occurred with clozapine (150 resp. 250 mg per day, the latter in combination with mirtazapine 60 mg/day) and one case with aripiprazole 15 mg in monotherapy. Decrease of the white blood count was detected after 14–21 days. Despite antibiotic treatment in all three cases and administration of G-CSF in one case, the ADR led to fatal pneumonia in one and fatal sepsis in two cases.

## Discussion

Neutropenia and agranulocytosis are rare, but potentially fatal ADRs of APDs. We evaluated 124 cases of neutropenia, 48 of which fulfilled the criteria of agranulocytosis. Accordingly, N&A occurred in 0.37 cases per 1000 inpatients treated with APDs, while agranulocytosis occurred in 0.14 cases per 1000 inpatients treated with APDs.

This study found that the incidence of N&A, but not agranulocytosis alone was higher among females in comparison to males. Some studies confirm this observation (Rawson et al. 1998; Shapiro et al. 1999; Théophile et al. 2004; van Staa et al. 2003), while other studies did not find any relevant sex-related differences (Strom et al. 1992). Female sex has been discussed as a susceptibility factor for drug-induced neutropenia (Grohmann et al. 1989; Tesfa et al. 2009). However, a recent study shows that the risk of agranulocytosis may be higher in middle-aged and younger females, whereas there is no sex difference among older individuals (Ibáñez et al. 2005). A number of studies analyzing drug-induced agranulocytosis have shown that the incidence increases with age (Rawson et al. 1998; Shapiro et al. 1999; Théophile et al. 2004). This may be due to the increasing number of drugs used and higher susceptibility for ADRs with increasing age. While these studies did not focus on APDs, there are also studies on clozapine-induced agranulocytosis that suggest a higher risk for older patients and females (Alvir et al. 1993; Copolov et al. 1998). Andrès et al. (Andrès et al. 2004) found that the incidence increases with age, as only 10% of cases were reported in children and young adults, and more than half of the cases of drug-induced agranulocytosis occurred in people over 60 years of age. They also conclude that the higher rate of agranulocytosis in women is likely to be biased because of the longer life expectancy, possibly resulting in longer periods of exposure to drugs (Strom et al. 1992). We did not find a significant correlation between the incidence of N&A and age in our sample.

Cases of N&A occurred significantly more often (*p* < 0.01) in patients treated with APDs suffering from schizophrenia, schizotypal and delusional disorders, and mania. This finding is not surprising, since these diagnoses represent the main indications for APDs that are generally used at higher dosages than in patients with, e.g., major depression or dementia. Moreover, the use of clozapine (i.e., the APD with the highest incidence of neutropenia/agranulocytosis) is limited to the treatment of therapy-resistant schizophrenia, and therefore expected not to be routinely used to treat other mental disorders.

In general, APD-induced N&A does not appear to be dose related with a few exceptions (i.e., quetiapine, risperidone and perazine). This can be interpreted as an indication of toxic cause aggravated by accumulation and not exclusively an immunological phenomena. On the other hand, we found cases of N&A under treatment with clozapine under relatively low daily dosages such as 75 or 100 mg. The mechanism of clozapine-induced N&A seems to have a mainly autoimmune background, rather than toxic, which seems to be supported by the data in our study, but the pathogenesis, despite multiple experiments, has not yet been fully elucidated (Wiciński and Węclewicz 2018).

As expected, most cases of agranulocytosis occurred under treatment with clozapine. Clozapine induces acute agranulocytosis in almost 1% of patients during the first 3 months of treatment (Alvir et al. 1993; Copolov et al. 1998). Literature shows that the risk decreases considerably after this initial period (Garbe 2007). A systematic review of case reports of non-chemotherapy drug-induced agranulocytosis revealed that the median duration of drug treatment before onset of acute agranulocytosis ranged between 2 days for dipyrone and 60 days for levamisole and was over 1 month for 71% of drugs (Andersohn et al. 2007). These findings are largely supported by our study, in which most cases of APD-induced N&A occurred during the first 3 months of treatment. However, physicians should remain aware of this ADR even after years of treatment, as the risk of APD-induced N&A does not fully subside.

This study’s observation that neutropenia was generally detected earlier than agranulocytosis, and that almost 60% of the agranulocytosis cases could be detected before the neutrophil count fell below < 0.1 × 10^9^ cells/L, implies that the monitoring that is standard nowadays is effective and potential damage can be recognized and averted at an early stage.

Following clozapine, the phenothiazine perazine showed the second highest incidence of N&A. In contrast, the other two chemical classes of first-generation APDs (i.e., butyrophenones and thioxanthines) were only rarely imputed at all and hardly ever imputed alone. In the publication “Agranulocytosis and significant leucopenia with neuroleptic drugs” from 1989 (Grohmann et al. 1989), using the predecessor of the AMSP database, seven cases of agranulocytosis were observed over a 9-year period in the psychiatric departments of the university hospitals in Munich and Berlin. One case occurred during treatment with clozapine in monotherapy, the other six during treatment with perazine. In three of these cases, perazine was used in monotherapy, one case occurred in combination with trimethoprim/sulfamethoxazole and in two cases in combination with tricyclic antidepressants. One previous publication using the AMSP database by Stübner et al. (2004) detected 63 events of APD-induced neutropenia and 22 cases of APD-induced agranulocytosis in a population of 122,562 patients between 1993 and 2000. Most changes in the WBC were attributed to clozapine (0.18% of patients exposed), carbamazepine (0.14%) and perazine (0.09%) (Stübner et al. 2004). Additionally, five cases of neutropenia during treatment with olanzapine and one case during treatment with risperidone were recorded. The confidence intervals of the incidence of APD-induced N&A reported by Stübner et al. were often wide and overlapping, therefore only providing a limited estimation of the incidence of N&A under individual APDs. The present study includes approximately twice as many events of APD-induced N&A, thus allowing for a more precise estimation and ranking of the risk of APD-induced N&A of specific APDs. However, the incidence of clozapine-induced agranulocytosis was unchanged at 0.15% of all patients exposed to clozapine in the present study.

The newer generation of clozapine-inspired second-generation APDs like quetiapine is generally considered to have reduced toxicity through improved potency, decreased dosage or structural modification (Li and Cameron 2012). However, potential toxic and/or immunological effects on the white blood cells need to be considered. Quetiapine may induce neutropenia more quickly than other drugs. This study includes a case of quetiapine-induced neutropenia manifesting after only 2 days of treatment with quetiapine. Moreover, quetiapine-induced neutropenia was detected after a median duration of only 16 days, which is remarkably shorter than the median time to onset of all other APDs. To the best of our knowledge, this observation has not been reported so far. AMSP previously reported on a fatal case of agranulocytosis in which quetiapine was used in monotherapy (Glocker et al. 2017). This was the first reported case of N&A occurring under quetiapine in monotherapy. Fryer and Billing ‘s (2020) case report of a quetiapine-induced neutropenia shows that even low dosages of quetiapine, in this case used for insomnia, can cause this potentially lethal ADR. These findings emphasize the importance of close monitoring not only of patients treated with clozapine, but also other APDs, especially quetiapine.

It needs to be emphasized that 102 of the 124 N&A cases (82.3%) occurred during combination therapy, mainly under the combination of two antipsychotic drugs, but also during combination therapy with non-psychiatric drugs. Because of the very low number of events of APD-induced neutropenia, this study is unable to identify specific high-risk combinations of APDs. Consequently, special vigilance should be exercised with any combination therapy that includes an antipsychotic drug with risk for N&A.

Research shows that fatality of drug-induced N&A has decreased over the past decades to about 5% owing to improved intensive care treatment and the availability of efficient broad-spectrum antibiotics (Andersohn et al. 2007). Mortality rate increases with age with higher frequency in people aged > 65 years; the incidence is also slightly higher in women up to 65 years of age (Ibáñez et al. 2005). Other features associated with increased mortality in drug-induced neutropenia are a neutrophil count < 0.1 × 10^9^ cells/l, concomitant renal disease, septicemia and shock (Johnston and Uetrecht 2015)*.*

An increased alertness of physicians and other health-care personnel to drug-induced agranulocytosis with prompt discontinuation of the suspected drug and treatment with hematopoietic cell growth factors have probably all contributed to the improved outcome of this potentially fatal ADR (Andersohn et al. 2007; Garbe 2007). Nevertheless, we still detected all in all three fatal cases in the observation period, reflecting a mortality of 2.4% of APD-induced neutropenia and 6.2% of APD-induced agranulocytosis. Interestingly, all of these patients were females > 65 years of age, which reflect the demographic risk factors of drug-induced N&A as mentioned above. Combination therapy, high starting dose (unknown in the aripiprazole case) and predisposition for drug-induced leucopenia should also be considered as potential risk factors.

### Strengths and limitations

AMSP is a structured drug surveillance program with a uniform documentation process. The 23-year observation period of more than 300,000 psychiatric inpatients treated with APDs enables the detection of rare ADRs with a low margin of error. Due to the inpatient setting, AMSP is able to assess actual drug utilization rates versus prescription rates, as is often the case in studies reflecting the outpatient setting. In controlled clinical trials, ADRs are described in a limited and defined population that may not adequately reflect a real world patient population. The AMSP data bank allows an estimation of the incidence of an ADR under real-world circumstances.

Compared to prospective randomized, placebo-controlled studies with healthy controls, the data obtained in this naturalistic setting have several limitations. Underreporting has to be taken into account, so that the incidence of N&A may be underestimated. The reporting of severe ADRs depends on clinicians that act as drug monitors in addition to their routine duties. Depending on their time and motivation as well as the staffing of the participating hospital, an individual and institutional bias in terms of underreported ADRs cannot be ruled out*.* Patients treated with APDs with a more well-known risk of N&A (e.g., clozapine) may have been more closely monitored for this ADR, therefore leading to a higher detection rate. Also, we only recorded cases of drug-induced N&A presenting during inpatient care. Data on ADRs that occur during the later course of treatment are only available if patients are re-admitted for example due to the occurrence of an ADR. Since polypharmacy and the imputation of more than one drug were included, the assessment of correlation and probability rating can be more difficult in some cases and may be more susceptible to errors.

## Conclusion

Overall, neutropenia and agranulocytosis are severe and potentially fatal complications that can occur during treatment with APDs. While some APDs are well known for their risk of inducing this ADR, the possible occurrence of N&A is less often considered under treatment with other ADRs. Therefore, the risk of APD-induced N&A should not be forgotten, even in the "post-clozapine" era. The results from this study largely agree with the currently available literature, highlighting the positive effects of alertness and established appropriate monitoring.

## Data Availability

All data analyzed during this study are included in this published article.
